# Diet Quality and Nutritional Value in Children and Adolescents with Excess Body Weight and Dyslipidemia Undergoing Low-Glycemic Index or Standard Diet

**DOI:** 10.3390/nu18030448

**Published:** 2026-01-29

**Authors:** Beata Bondyra-Wiśniewska, Anna Harton

**Affiliations:** Department of Dietetics, Institute of Human Nutrition Sciences, Warsaw University of Life Sciences (WULS), 159C Nowoursynowska St, 02-776 Warsaw, Poland; anna_harton@sggw.edu.pl

**Keywords:** diet quality, nutritional value, DQI, glycemic index, childhood obesity, adolescents, dyslipidemia

## Abstract

**Background/Objectives**: The increasing problem of excess body weight and the resulting dyslipidemia among children and adolescents is a serious health challenge that may have long-term consequences. In this context, the search continues for an optimal diet that will support both body weight normalization and improvement in lipid parameters. In the prevention and treatment of cardiovascular diseases and lipid disorders, limiting highly processed foods and replacing them with natural, minimally processed options lower in fat, saturated fatty acids (SFA), cholesterol, sugar, and salt is recommended. This study aimed to assess the quality and nutritional value of the low-glycemic index (LGI) diet and standard (ST) diet. **Methods**: Both diets were based on the principal recommendation of the Cardiovascular Health Integrated Lifestyle Diet-2 (CHILD-2). The Diet Quality Index (DQI) was used to assess the diet quality. Moreover, the nutritional value of the diet was assessed before and after 8 weeks of intervention. The study included 40 patients aged 8–16 years with excess body weight and dyslipidemia who completed the entire nutritional intervention. **Results**: This study demonstrated that both LGI and ST diets were effective in improving diet quality. The nutritional intervention led to an almost 2-fold reduction in the mean DQI score in the LGI diet group and almost 1.5-fold reduction in the ST diet group (significant differences between groups). No participants were classified into a lower diet quality category than at baseline. The percentage of participants with minimum moderate diet quality reached 100% in the LGI diet group and 44% in the ST diet group. Changes in nutritional value were similar in the LGI and ST diet groups. Both interventions resulted in a significant increase in protein and fiber consumption, as well as a decrease in cholesterol, SFA, and sodium. A greater improvement in diet quality was significantly associated with lower fat and SFA, as well as higher vitamin C intake, in both groups; specifically, it was also associated with reduced cholesterol and sugar intake in the LGI diet group, and reduced sodium intake in the ST diet group. **Conclusions**: These results suggest that in the dietary therapy of children and adolescents with excess body weight and dyslipidemia, the quality of the diet is crucial, as it is associated with beneficial changes in nutritional value, which may have a positive impact on patient health. To achieve this, however, constant and consistent cooperation with a dietitian is necessary to help implement appropriate dietary recommendations in practice. Further long-term, larger-scale studies are needed.

## 1. Introduction

The increasing prevalence of excess body weight among children worldwide and related metabolic disorders, including dyslipidemia, poses a serious public health challenge [1,2]. According to the World Health Organization (WHO), in 2022, more than 390 million children and adolescents aged 5 to 19 were overweight, including 160 million with obesity [3]. This means that over the years 1990–2022, the prevalence of excess body weight in this age group increased from 8 to 20%. Excess body weight at a young age is associated with many serious health consequences, including atherosclerosis, hypertension, type 2 diabetes, and metabolic syndrome, which are traditionally associated with adulthood [4,5]. For this reason, early diagnosis and implementation of effective nutritional strategies in childhood, rather than the need for long-term treatment of diseases in adulthood, are crucial [6,7].

A healthy diet plays a crucial role in managing excess body weight and the associated dyslipidemia [8]. Extremely restrictive and nutrient-poor diets should be avoided, especially in the pediatric population. The primary goal is to enhance health and cultivate healthy habits that will endure a lifetime [9,10]. It is especially important that the diet is based on healthy eating principles, supplemented by specific guidelines such as the CHILD-2 recommendations (e.g., limit fat intake < 30% of energy, saturated fatty acids (SFA) to <7% of energy, trans fatty acids, cholesterol to <200 mg, and sugar, increase the intake of polyunsaturated fatty acids and fiber) [11]. Due to the multitude of dietary recommendations and standards, various indicators have been developed to assess dietary quality based on selected nutrients and food groups [12]. From among these tools, the Diet Quality Index (DQI) was selected for this study, as it specifically evaluates these key nutritional elements [13].

The main aim of this article was to assess the quality and nutritional value of two types of diet (low-glycemic index (LGI) diet and standard (ST) diet) used by children and adolescents with excess body weight and dyslipidemia. Additionally, the study assessed the correlation between the change in DQI scores after 8 weeks of intervention and selected nutrients. The obtained data can be used to design nutritional interventions for children and adolescents with overweight/obesity and dyslipidemia.

## 2. Materials and Methods

The study was conducted in 2019–2020 among 64 children and adolescents aged 8–16 living in Poland, in the Mazowieckie Voivodship. Data from 40 participants (24 boys and 16 girls) who completed the study were included in the analysis, representing a retention rate of 62.5%. The main reasons for dropping out of the study included non-compliance with dietary recommendations and problems with making meals. These patients were from the Children’s Memorial Health Institute in Warsaw and were qualified to participate in the nutritional intervention program by a pediatrician based on a medical interview. All participants were diagnosed with overweight or obesity and dyslipidemia. Body weight was measured using the TANITA MC-780 P MA (TANITA Corporation, Tokyo, Japan) professional multi-frequency body composition analyzer (accuracy: 100 g). Body height was measured using a stadiometer in a standing anthropometric position. All measurements were performed with participants wearing light clothing and without shoes, headwear, hair ornaments, or heavy items in pockets (e.g., phones, wallets). Then, the body mass index (BMI) was calculated using the following formula: BMI [kg/m^2^] = body mass [kg]/(body height [m])^2^. The BMI values were compared with Polish reference values for the assessment of growth and nutritional status in children and adolescents aged 3–18 years [14]. According to the International Obesity Task Force criteria [15], overweight was defined as a BMI between the 85th and 95th percentile, and obesity as a BMI above the 95th percentile for age and gender. Dyslipidemia was defined as the presence of at least 1 lipid abnormality, such as high total cholesterol, high low-density lipoprotein cholesterol, high triglycerides, or low high-density lipoprotein cholesterol, according to the American College of Cardiology [16]. Participants did not use any pharmaceuticals, dietary supplements, or nutraceuticals affecting lipid metabolism, blood pressure, or body weight. The study protocol was approved by the Ethics Committee of the Faculty of Human Nutrition and Consumer Science, Warsaw University of Life Sciences WULS, Poland (10p/2017, 17 May 2017). Additional information about the design and study is provided in the study protocol [17].

Patients were randomly selected and allocated to one of the two groups: the intervention group with a low-glycemic index (LGI) diet or the control group with a standard (ST) diet. Allocation was single-blind—participants did not know which group they were assigned to. Both diets were based on the principal recommendation of the Cardiovascular Health Integrated Lifestyle Diet-2 (CHILD-2) [11]. The main goal of the CHILD-2 guidelines is to limit the intake of fat (<30% of energy), SFA (<7% of energy), trans fatty acids, cholesterol (<200 mg), and sugar, as well as to increase the intake of polyunsaturated fatty acids and fiber. The LGI diet was additionally based on low-glycemic index products (<55), including whole-grain products, low-starch vegetables, and raw fruit with a reduced content of sugars, nuts, and seeds [18]. Using glycemic index (GI) values from international tables [19], the glycemic load (GL) was calculated according to the formula: GL = available carbohydrates [g] × GI/100. The total daily GL represented the sum of the glycemic loads of all consumed foods. The diets were classified based on the daily GL: <120 for the LGI diet and ≥120 for the ST diet. The energy value of the diets was adjusted individually according to the degree of excess body weight, taking into account the basal metabolic rate and physical activity level. Both diets were nutritionally balanced and matched for macronutrient composition: 15–20% from protein and 50–60% from carbohydrates for daily energy. As part of the study, participants and their primary caregivers attended three visits with a dietician: a baseline visit, a control visit after 4 weeks, and a final visit after 8 weeks from the beginning of the study. These visits included anthropometric measurements, analysis of food intake records to verify patients’ adherence to the diet, nutritional education, and assessment of physical activity levels.

Data on current and habitual food intake were collected and analyzed by a dietitian. Parents were responsible for maintaining children’s nutritional records. To assess current food intake before the start of the nutritional intervention program, a 3-day food record was used. Patients receive a template and instructions for completing it. They were asked to carefully record all food and drinks they consumed on 2 weekdays and 1 day off from school. Patients also kept food records throughout their participation in the nutritional intervention program. The dietitian calculated nutritional values from the food records using a table of nutritional values for food products and dishes [20]. Data on habitual food intake of products from 8 food groups, including sweets and snacks, dairy products and eggs, cereal products, fats, fruits, vegetables and seeds (e.g., pumpkin, sesame, sunflower, wheat germ), meat products and fish, and beverages, were also collected using the validated Food Frequency Questionnaire (FFQ-6) [21]. Data using the FFQ-6 questionnaire were collected before and after participation in the nutritional intervention program. Data on current and habitual food intake were used to assess diet quality. The quality of diets was assessed using the DQI [13], which includes eight parameters: the share of energy from total fats and saturated fatty acids (SFA), the intake of cholesterol, protein, calcium, and sodium, the consumption of vegetables/fruits, and whole-grain cereal-based products. For each of the assessed parameters, points of 0, 1, or 2 were awarded, with 0 reflecting compliance with dietary recommendations and 2 reflecting non-compliance. A lower score indicates better diet quality. Interpretation of the obtained score is possible after classification into one of five classes defining the quality of the diet: unsatisfactory (11–16 points), acceptable (8–10), moderate (6–7), good (4–5), or high (0–3). The study adopted modified recommendations for SFA and cholesterol intake, based on the CHILD-2 guidelines [11]. Intake of total protein in g/kg healthy body weight was expressed as % of RDA by gender and age [22]. For sodium and potassium, the adopted values were in accordance with the dietary guidelines for the Polish population by gender and age [22].

All data, including both nutritional value and diet quality, were evaluated before the study and after 8 weeks of the nutritional intervention.

### Statistical Analysis

All statistical analyses were conducted using Statistica version 13.1 (Copyright©StatSoft, Inc., 1984–2014, Cracow, Poland). For all tests, *p* < 0.05 was considered significant. The Shapiro–Wilk test was used to assess the normality of distributions. Nonparametric tests were used in statistical analyses due to the lack of normal distribution in groups.

The Wilcoxon matched-pairs test and Mann–Whitney U test were used to evaluate quantitative data. The Wilcoxon matched-pairs test was used to compare the values of the DQI, nutritional value, and glycemic load (GL) before (baseline) and after 8 weeks of the nutritional intervention program. The Mann–Whitney U test was used to compare the differences in the DQI and the change (∆) in the levels of the nutritional value and GL between the LGI and ST diet groups. Spearman’s correlation was used to assess the association between the change in DQI scores and selected components of nutritional value. The change was the difference in values between the data obtained after 8 weeks and the baseline data. All quantitative data are expressed as mean ± standard deviation (SD). Statistical tests used for analysis are described separately under each data table. All data are presented for the total group and by type of diet used.

## 3. Results

### 3.1. Sample Description

In total, 40 children and adolescents with excess body weight and dyslipidemia completed the study. Patients who were overweight constituted 40% (*n* = 16) of the respondents, and 60% (*n* = 24) were diagnosed with obesity. The mean age of the participants was 13.35 ± 2.63 years.

### 3.2. Assessment of Diet Quality

Table 1 shows the percentage distribution of the participants at baseline and after 8 weeks of the nutritional intervention program, according to the compliance of individual assessed components with the DQI scoring. Data for the total group are presented in Appendix A (Table A1).

Before the intervention in both study groups, the parameters determining low diet quality were most often a high share of energy from SFA, high cholesterol intake, low calcium intake, and insufficient consumption of whole-grain cereal products. In turn, the recommendation for ≥5 servings of vegetables and fruits per day was the most frequently achieved. The applied nutritional intervention contributed to more frequent adherence to recommendations for lower intake of SFA (saturated fatty acids), cholesterol, and sodium, as well as higher calcium intake in both groups. In the LGI diet group, an additional factor influencing better diet quality was the more frequent consumption of a minimum of 6 servings of whole-grain cereal-based products.

Table 2 presents the distribution of participants across diet quality categories after 8 weeks of the nutritional intervention program. All participants whose diet quality was initially unsatisfactory (*n* = 26) progressed to acceptable (*n* = 4), moderate (*n* = 18), or good (*n* = 4). After the nutritional intervention, no participant was classified into a lower diet quality category than at baseline. Data for the total group are presented in Appendix A (Table A2).

Table 3 presents the mean DQI scores at baseline and after 8 weeks of nutritional intervention. The mean DQI score in the LGI diet group was almost twice as low after 8 weeks of intervention compared to baseline, and almost 1.5 times lower in the ST diet group. The applied nutritional intervention improved diet quality significantly more in the LGI diet group compared to the ST diet group. Data for the total group are presented in Appendix A (Table A3).

### 3.3. Assessment of the Nutritional Value

Table 4 presents the baseline nutritional value and GL of the diets, as well as the relationship between the percentage change in nutrients and GL after the 8-week nutritional intervention. Data for the total group are presented in Appendix A (Table A4).

In the LGI diet group, the nutritional intervention resulted in increased intake of protein, *n*-3, and fiber. In turn, the content of fat, cholesterol, and SFA, as well as GL, decreased. In the ST diet group, after 8 weeks of nutritional intervention, the intake of protein and fiber increased, while the level of cholesterol, SFA, sugars, and GL decreased. Changes in nutritional value following the dietary intervention were similar between the LGI and ST diet groups. However, significant differences were demonstrated in GL, with a greater reduction observed in the LGI diet group (Table 4).

Table 5 shows the baseline mineral levels and their percentage change after the 8-week nutritional intervention. Data for the total group are presented in Appendix A (Table A5).

In both the LGI and ST diet groups, the nutritional intervention resulted in a significant increase in the intake of potassium, calcium, phosphorus, magnesium, iron, and zinc, while the level of sodium decreased. Phosphorus and magnesium intake increased more in the LGI compared to the ST diet group.

Table 6 shows the baseline vitamin levels and their percentage change after the 8-week nutritional intervention. Data for the total group are presented in Appendix A (Table A6).

In both the LGI and ST diet groups, the nutritional intervention resulted in a significant increase in the intake of vitamins A, E, C, and B, except B_12_ in the ST diet group. Vitamin intake did not decrease in any of the groups. No significant differences were observed between the LGI and ST groups in the levels of selected vitamins after 8 weeks of nutritional intervention.

### 3.4. DQI Score and Nutritional Value

Correlations between the change in DQI scores after 8 weeks of intervention and selected components of nutritional value are presented in Table 7. A greater improvement in diet quality was significantly associated with lower energy, total fat, and SFA intake, as well as higher vitamin C intake in both study groups. Additionally, in the LGI diet group, a greater improvement in diet quality was associated with reduced intake of protein, carbohydrates, cholesterol, and sugars, whereas in the ST diet group, it was associated with a significant reduction in sodium intake. Data for the total group are presented in Appendix A (Table A7).

## 4. Discussion

This study assessed the quality and nutritional value of two types of diet: low-glycemic index (LGI) diet and standard (ST) diet used by children and adolescents with excess body weight and dyslipidemia.

Diet quality was assessed using the Diet Quality Index (DQI) [13]. At baseline, the mean DQI score was 11.64 ± 1.56 for the LGI diet group and 10.78 ± 2.53 for the ST diet group, with 82% and 44% of participants, respectively, classified as having an “unsatisfactory quality diet”. These results are consistent with a recent Polish study in the pediatric population, which observed significantly lower diet quality in individuals with overweight and obesity compared to their normal weight peers [23,24]. Additionally, minimum a 40% of the participants showed a low-quality diet [23,24]. In another study, over 80% of Polish children and adolescents aged 10–16 had a low level of intensity of pro-health features [25]. A general decline in diet quality among children and adolescents has been observed globally [26,27]. Studies using comprehensive indicators often reveal that a large percentage of youth do not meet dietary recommendations [28]. For instance, broad analyses of diet quality in US children and adolescents have shown that overall mean scores remain suboptimal, with a high intake of energy from low-quality components [29,30]. Similarly, studies in European populations have found that a significant majority of children often have diets classified as low-quality or requiring improvement [31,32,33,34,35,36,37,38]. Among 1335 participants aged 2–18 years, children and adolescents with overweight or obesity were observed to have poor diet quality, with children with overweight having better diet quality compared to children with obesity [39]. Some researchers have confirmed a correlation between lower diet quality and higher BMI values [40,41]. On the other hand, an analysis of data from 15,658 participants aged 2–19 years found that better diet quality was associated with a lower risk of overweight and obesity [42]. In American youth aged 8–15, higher scores on diet quality indices were associated with lower BMI gains both in the current period and over the next 2–3 years, confirming the long-term benefits of a high-quality diet [43]. In adults, it was observed that a ten-year improvement in diet quality was associated with smaller weight gain, suggesting that maintaining a high-quality diet and improving diet quality over time may prevent excessive weight gain [44]. Systematic reviews and meta-analyses also confirm the role of high-quality dietary patterns in the prevention of abdominal obesity and metabolic syndrome [41]. In contrast, in children whose diet was based on processed and sweet products, the most unfavorable changes in adipose tissue mass and abdominal fat were observed [45]. A systematic review of 128 studies involving children and adolescents aged 2–17 years showed that greater adherence to balanced dietary patterns was associated with lower body fat, waist circumference, blood pressure, and metabolic risk [46]. Despite ample evidence of those associations, some cross-sectional studies have not shown a statistically significant correlation between overall diet quality scores and body mass [33,46,47]. Nevertheless, higher diet quality scores were associated with several other positive habits, such as eating more fruit and vegetables, avoiding chips and sweets, drinking water regularly, not skipping meals, and eating breakfast every day [47,48]. Furthermore, lower overall diet quality scores are more frequently observed among children with obesity compared to children with a normal body weight [24,33].

In our 8-week nutritional intervention program for children and adolescents with excess body weight and dyslipidemia, we achieved significant improvements in diet quality compared to baseline, with significantly greater improvements observed in the LGI diet group compared to the ST diet group. The mean DQI score decreased almost 2-fold in the LGI diet group and almost 1.5-fold in the ST diet group, with the percentage of participants achieving at least moderate diet quality at 100% and 44%, respectively. Furthermore, the transition of all participants from the “unsatisfactory quality diet” class to the higher classes was a very positive result. A retrospective study of 157 children with primary hyperlipidemias showed improvement in diet quality scores after 6 months of nutritional counseling in 65% of patients, but only 33.8% progressed to a higher dietary adherence class [49]. In our study, such a change in the DQI was mainly associated with a higher percentage of participants meeting the recommended intake of total fat, SFA, and cholesterol. Importantly, this improvement was observed in both the LGI and ST diet groups. Additionally, in the LGI diet group, the consumption of whole-grain cereal-based products increased compared to the ST diet group, which resulted from the specificity of the study and dietary assumptions. These results are consistent with more recent publications that demonstrate that brief, well-designed interventions can significantly improve overall diet quality scores [50]. In the 2-month randomized ALINFA study conducted in Spain, a nutritional intervention focused on improving diet quality, implemented in children aged 6–12 years, resulted in a significant increase in the mean dietary quality index score (*p* < 0.001). The intervention also improved the nutritional profile, including increased consumption of whole grains, legumes, and nuts, and reduced consumption of sweets and fast food [51]. Similarly, another dietary intervention also led to an increase in diet quality scores in children aged 7–16 years with abdominal obesity, both in the standard care group and in the group recommending a specific, high-quality, energy-reduced diet [52]. Also, studies evaluating school-based interventions in children have found significant improvements in mean dietary quality scores post-intervention [53,54]. Maintaining a high-quality diet from early childhood plays a crucial role in establishing positive eating habits, which in turn leads to better diet quality later in life [55]. Data from 2017 demonstrate that low dietary quality—and especially major risk factors like excess sodium intake and insufficient whole-grain cereal-based products and fruits—was the leading cause of 11 million deaths and the loss of 255 million disability-adjusted life years (DALYs) globally [26].

Assessing the quality of a diet without reference to its nutritional value seems incomplete; therefore, the nutritional value of the diets was also assessed. Both examined groups showed a significant reduction in cholesterol, SFA, and sodium intake, as well as an increase in protein, fiber, mineral, and vitamin intake. Changes in most nutrients after the dietary intervention were similar in the LGI and ST diet groups. However, a greater reduction in GL was observed in the LGI diet group, demonstrating that patients adhered to the dietary recommendations. Furthermore, improved diet quality correlated significantly with lower fat and SFA intake in both groups, indicating a reduction in animal-based food sources in favor of plant-based options. Additionally, the better diet quality was associated with increased vitamin C intake, indicating a higher consumption of fruits and vegetables as another beneficial post-intervention change in both diets. Furthermore, improved diet quality was associated with reduced cholesterol and sugar intake in the LGI diet group, whereas in the ST diet group, it was associated with lower sodium intake. The ALINFA study showed that increasing dietary quality resulted in a reduction in the intake of calories, total fat, and SFA, as well as an increase in fiber intake [51]. The SENDO project demonstrated that better diet quality reduces the risk of deficiency of ≥3 micronutrients [56]. Similar results were obtained among Israeli adolescents—higher diet quality was associated with a better micronutrient profile and negatively influenced the energy density of the diet [57]. In children and adolescents with abdominal obesity, better results of diet quality indicators were associated with lower micronutrient deficiency [52]. Research conducted in Spain also shows that children with better diet quality consume less ultra-processed food (UPF) [58]. This inverse relationship has been quantified in various studies. For example, in the preschool group, it was observed that every two additional points in the Mediterranean Diet Quality Index (KIDMED) score were associated with a 3% lower energy intake from UPF [59]. In a study of US children, higher consumption of UPF was directly associated with lower diet quality scores [60]. It was observed that as the intake of calories from UPF increased, the consumption of healthy foods decreased, and the consumption of unhealthy foods increased.

A well-planned nutritional intervention will simultaneously support healthy weight loss and cardiovascular health in children and adolescents [61]. However, to effectively change the quality of a diet, its basic assumptions, i.e., quantitative guidelines, are crucial. Such a diet should be based particularly modified fatty acids—primarily reduced amounts of SFA, trans fatty acids, cholesterol, and sugar, and increased amounts of polyunsaturated fatty acids and fiber. These assumptions were included in recommendations CHILD-2 and formed the basis of the planned dietary therapy in our study. However, even the best nutritional model should be part of a broader lifestyle change. Regular physical activity, involvement and support of the entire family, specialist care (including a pediatrician and dietitian), and an adequate amount of sleep significantly increase the chance of reducing body weight and improving the lipid profile, as well as maintaining the effects over time [62,63].

### Strengths and Limitations

This study has several significant strengths. Firstly, focusing on a clearly defined risk group, i.e., children and adolescents with overweight or obesity and dyslipidemia, is of great clinical importance. This approach is supported by evidence from meta-analysis, which shows greater effectiveness of intervention in groups of children with overweight and obesity compared to the general population [64]. Secondly, the use of the DQI, commonly used to assess dietary quality, is a significant methodological advantage. In our research, in addition to analyzing eating behavior, we also assessed the nutritional value of the diet. This comprehensive approach to dietary analysis allowed us to relate changes in dietary quality to changes in the consumption of individual nutrients and monitor the effectiveness of dietary changes.

Although our study provides valuable data on the effectiveness of a nutritional intervention, its methodological limitations, which are typical of intervention studies in the pediatric population, should be considered. Firstly, there was a significant dropout rate (62.5% retention), the main reason for which was non-compliance with dietary recommendations and problems with meal making. The smaller sample size (*n* = 40) significantly limits the statistical power and generalizability of the results. Recruiting and retaining participants in pediatric clinical trials is a common challenge, often leading to small sample sizes and selection bias. Secondly, the duration of the intervention, at only 8 weeks, is relatively short. A review of thematic literature indicates that changes in anthropometric parameters, including BMI, may require at least 6 months to become significant [65]. Further follow-up studies should be conducted to assess the durability of the habits developed and their long-term impact on clinical indicators. Another limitation is the reliance on self-report data to assess dietary quality. Although this method is widely used, it is prone to error because it relies on self-reported data rather than objective measurements. Consequently, this can lead to underestimation or overestimation of food intake. On the other hand, self-report data allow for detailed information on food and beverage consumption and monitoring the effectiveness of dietary interventions. Furthermore, maintaining such records can increase patients’ motivation to change. Moreover, the dietitian was responsible for the dietary interview, intervention, and nutritional education throughout the entire course of dietary therapy; therefore, they could not be blinded. This, in turn, could potentially influence the obtained results. However, to minimize this error, standardized methods of intake assessment (e.g., FFQ-6) were employed, and objective anthropometric measurements were also relied upon. It should be noted that the statistical analyses did not address the issue of multiple comparisons due to the exploratory nature of some secondary analyses. The purpose of this approach was to determine whether there were potential associations worth investigating further in the future. Therefore, the results regarding differences between the LGI and ST diets, especially those whose significance was close to the 0.05 threshold, should be interpreted with caution.

## 5. Conclusions

Our study showed that the LGI and ST diets were effective in improving diet quality as measured by the DQI in children and adolescents with overweight or obesity and dyslipidemia. Notably, a greater improvement in diet quality was associated with lower fat and SFA intake in both groups. These results suggest that in the dietary therapy of children and adolescents with excess body weight and dyslipidemia, the quality of the diet is crucial, as it is associated with beneficial changes in nutritional value, which may have a positive impact on patient health. To achieve this, however, constant and consistent cooperation with a dietitian is necessary to help implement appropriate dietary recommendations in practice. This short-term, intensive nutritional intervention showed that when choosing a diet, the key factor may not be the type of diet itself, but the actual improvement in the quality of nutrition. Further research is needed to confirm and expand on our findings. Similar interventions with larger groups and for a longer period (at least 6 months) are recommended to assess the sustainability of dietary changes and their long-term impact on anthropometric and cardiometabolic parameters. Furthermore, to gain a more complete picture of the relationship between diet and health in children and adolescents, a holistic approach is recommended, considering other lifestyle factors such as sleep quality, physical activity levels, and screen time.

## Figures and Tables

**Table 1:** Assessment of selected components of DQI (% of participants).

Variable	Intake	Points	LGI Diet (*n* = 22)		ST Diet (*n* = 18)	
			Baseline	After 8 Weeks	Baseline	After 8 Weeks
Fats (% of energy)	≤30	0	18	36	44	89
	30–40	1	73	64	56	11
	>40	2	9	0	0	0
SFA ^1^ (% of energy)	≤7	0	0	18	22	56
	7–10	1	0	82	11	44
	>10	2	100	0	67	0
Cholesterol ^1^ (mg/day)	≤200	0	9	18	0	11
	200–300	1	36	82	33	89
	>300	2	55	0	67	0
Protein ^2^ (% of RDA)	≤100	0	0	0	0	0
	100–150	1	9	0	22	0
	>150%	2	91	100	78	100
Sodium ^3^ (% of AI)	≤100	0	10	18	22	22
	100–150	1	45	82	22	67
	>150%	2	45	0	56	11
Calcium ^3^ (% of RDA)	≥100	0	0	9	0	0
	67–99	1	9	82	22	89
	<67	2	91	9	78	11
Whole-grain cereal-based products (portions/day)	≥6	0	5	100	0	0
	4–5	1	0	0	0	0
	0–3	2	95	0	100	100
Vegetables and fruits (portions/day)	≥5	0	91	100	78	100
	3–4	1	0	0	11	0
	0–2	2	9	0	11	0

LGI diet—low-glycemic index diet; ST diet—standard diet; SFA—saturated fatty acids; RDA—Recommended Dietary Allowance; AI—Adequate Intake; ^1^ adopted recommendations for CHILD-2 [11]; ^2^ intake of total protein in g/kg healthy body weight expressed as % of RDA by gender and age [22]; ^3^ adopted values in accordance with the dietary guidelines for the Polish population by gender and age [22].

**Table 2:** Participant distribution (%) across diet quality categories at baseline and after 8 weeks according to the DQI score.

	Variable	LGI Diet (*n* = 22)		ST Diet (*n* = 18)	
Diet Quality *		Baseline	After 8 Weeks	Baseline	After 8 Weeks
Unsatisfactory		82	0	44	0
Acceptable		18	0	44	56
Moderate		0	73	12	44
Good		0	27	0	0

LGI diet—low-glycemic index diet; ST diet—standard diet; * the “high” category was not included in the table because no participants met this criterion.

**Table 3:** Mean DQI score before and after nutritional intervention (mean ± SD).

Variable	LGI Diet (*n* = 22)		ST Diet (*n* = 18)		*p*-Value
	Baseline	After 8 Weeks	Baseline	After 8 Weeks	
DQI	11.64 ± 1.56	6.09 ± 1.02	10.78 ± 2.53	7.44 ± 0.98	<0.001 ^†^ 0.002 ^‡^ ns ^a^ <0.001 ^b^

DQI—Diet Quality Index; LGI diet—low-glycemic index diet; ST diet—standard diet; ns—not significant (*p* ≥ 0.05); significant differences between baseline and after 8 weeks (Wilcoxon matched-pairs test) in the following groups (*p* < 0.05): ^†^ LGI, ^‡^ ST; significant differences between LGI diet and ST diet (Mann–Whitney U Test) at the following time points (*p* < 0.05): ^a^ baseline, ^b^ after 8 weeks.

**Table 4:** Change (∆ %; mean ± SD) in the nutritional value and glycemic load of the diet after 8 weeks of nutritional intervention.

Variable		LGI Diet (*n* = 22)	ST Diet (*n* = 18)	*p*-Value
Energy (kcal/day)	Baseline	2355.59 ± 686.20	2151.89 ± 346.75	ns ^†^ ns ^‡^ ns ^a^
	∆ %	0.44 ± 27.50	1.33 ± 20.65	
Protein (g/day)	Baseline	96.52 ± 31.10	91.51 ± 17.86	0.006 ^†^ 0.001 ^‡^ ns ^a^
	∆ %	28.32 ± 34.40	20.28 ± 20.53	
Fats (g/day)	Baseline	88.35 ± 25.85	72.97 ± 16.50	0.024 ^†^ ns ^‡^ ns ^a^
	∆ %	−7.52 ± 29.50	0.58 ± 28.72	
Carbohydrates (g/day)	Baseline	306.19 ± 91.43	292.01 ± 47.07	ns ^†^ ns ^‡^ ns ^a^
	∆ %	−4.10 ± 44.24	−11.13 ± 32.86	
Cholesterol (mg/day)	Baseline	366.30 ± 158.01	379.31 ± 102.57	<0.001 ^†^ <0.001 ^‡^ ns ^a^
	∆ %	−26.41 ± 29.46	−36.45 ± 22.23	
SFA (g/day)	Baseline	34.41 ± 9.63	25.81 ± 9.09	<0.001 ^†^ 0.006 ^‡^ ns ^a^
	∆ %	−39.57 ± 17.72	−21.42 ± 36.50	
MUFA (g/day)	Baseline	32.86 ± 11.16	28.50 ± 5.07	ns ^†^ ns ^‡^ ns ^a^
	∆ %	3.83 ± 39.22	4.77 ± 23.61	
PUFA (g/day)	Baseline	14.03 ± 6.84	12.65 ± 5.29	ns ^†^ ns ^‡^ ns ^a^
	∆ %	25.70 ± 68.19	9.24 ± 37.58	
*n*-3 (g/day)	Baseline	2.24 ± 1.27	2.28 ± 1.94	0.003 ^†^ ns ^‡^ ns ^a^
	∆ %	95.09 ± 114.10	82.98 ± 99.25	
*n*-6 (g/day)	Baseline	11.78 ± 6.00	10.35 ± 4.57	ns ^†^ ns ^‡^ ns ^a^
	∆ %	14.65 ± 61.07	0.46 ± 28.97	
Fiber (g/day)	Baseline	25.00 ± 9.49	19.60 ± 4.42	<0.001 ^†^ <0.001 ^‡^ ns ^a^
	∆ %	77.84 ± 61.14	68.34 ± 44.45	
Sugars (g/day)	Baseline	87.49 ± 40.60	88.31 ± 29.08	ns ^†^ 0.039 ^‡^ ns ^a^
	∆ %	−4.10 ± 44.24	−11.13 ± 32.86	
Glycemic Load	Baseline	146.56 ± 57.19	152.84 ± 28.79	<0.001 ^†^ 0.010 ^‡^ 0.006 ^a^
	∆ %	−32.73 ± 23.49	−12.79 ± 20.73	

SFA—saturated fatty acids; MUFA—monounsaturated fatty acids; PUFA—polyunsaturated fatty acids; LGI diet—low-glycemic index diet; ST diet—standard diet; ns—not significant (*p* ≥ 0.05); significant differences between baseline and after 8 weeks (Wilcoxon matched-pairs test) at the following groups: ^†^ LGI diet, ^‡^ ST diet; ^a^ significant differences in ∆ % between LGI and ST diet groups (Mann–Whitney U Test; *p* < 0.005).

**Table 5:** Change (∆ %; mean ± SD) in the mineral levels after 8 weeks of nutritional intervention.

Variable		LGI Diet (*n* = 22)	ST Diet (*n* = 18)	*p*-Value
Sodium (mg/day)	Baseline	2295.82 ± 985.63	2145.33 ± 752.21	0.004 ^†^ 0.014 ^‡^ ns ^a^
	∆ %	−16.41 ± 39.46	−12.35 ± 39.78	
Potassium (mg/day)	Baseline	3314.26 ± 964.20	3339.54 ± 533.24	<0.001 ^†^ <0.001 ^‡^ ns ^a^
	∆ %	54.53 ± 43.03	39.09 ± 16.86	
Calcium (mg/day)	Baseline	660.81 ± 246.78	719.80 ± 212.79	<0.001 ^†^ 0.001 ^‡^ ns ^a^
	∆ %	92.38 ± 100.06	53.77 ± 64.40	
Phosphorus (mg/day)	Baseline	1346.56 ± 394.30	1354.32 ± 208.76	<0.001 ^†^ <0.001 ^‡^ 0.035 ^a^
	∆ %	64.21 ± 49.31	34.10 ± 19.96	
Magnesium (mg/day)	Baseline	294.13 ± 88.02	293.81 ± 56.84	<0.001 ^†^ <0.001 ^‡^ <0.001 ^a^
	∆ %	97.14 ± 67.43	45.55 ± 23.57	
Iron (mg/day)	Baseline	12.17 ± 3.88	11.24 ± 2.53	<0.001 ^†^ <0.001 ^‡^ ns ^a^
	∆ %	48.91 ± 47.52	36.56 ± 27.34	
Zinc (mg/day)	Baseline	29.18 ± 34.03	21.89 ± 27.47	0.004 ^†^ 0.010 ^‡^ ns ^a^
	∆ %	11.57 ± 3.53	10.44 ± 2.59	

LGI diet—low-glycemic index diet; ST diet—standard diet; ns—not significant (*p* ≥ 0.05); significant differences between baseline and after 8 weeks (Wilcoxon matched-pairs test) at the following groups: ^†^ LGI diet, ^‡^ ST diet; ^a^ significant differences in ∆ % between LGI and ST diet groups (Mann–Whitney U Test; *p* < 0.005).

**Table 6:** Change (∆ %; mean ± SD) in the vitamin levels after 8 weeks of nutritional intervention.

Variable		LGI Diet (*n* = 22)	ST Diet (*n* = 18)	*p*-Value
Vitamin A (μg/day)	Baseline	1297.91 ± 502.22	1075.51 ± 434.90	<0.001 ^†^ <0.001 ^‡^ ns ^a^
	∆ %	81.87 ± 67.65	124.88 ± 83.78	
Vitamin E (mg/day)	Baseline	13.15 ± 5.56	11.63 ± 4.99	<0.001 ^†^ 0.004 ^‡^ ns ^a^
	∆ %	84.03 ± 80.14	83.92 ± 76.01	
Thiamine (mg/day)	Baseline	1.54 ± 0.42	1.32 ± 0.35	0.006 ^†^ 0.006 ^‡^ ns ^a^
	∆ %	26.55 ± 31.59	33.82 ± 30.74	
Riboflavin (mg/day)	Baseline	1.74 ± 0.61	1.84 ± 0.47	<0.001 ^†^ 0.002 ^‡^ ns ^a^
	∆ %	54.70 ± 45.21	43.08 ± 34.55	
Niacin (mg/day)	Baseline	19.02 ± 6.85	17.69 ± 5.28	<0.001 ^†^ 0.001 ^‡^ ns ^a^
	∆ %	58.22 ± 43.89	51.52 ± 44.89	
Vitamin B_6_ (mg/day)	Baseline	2.07 ± 0.67	2.21 ± 0.57	<0.001 ^†^ <0.001 ^‡^ ns ^a^
	∆ %	78.72 ± 52.63	56.69 ± 35.91	
Folates (μg/day)	Baseline	317.04 ± 145.62	313.89 ± 88.06	<0.001 ^†^ <0.001 ^‡^ ns ^a^
	∆ %	116.14 ± 88.09	87.21 ± 36.52	
Vitamin B_12_ (μg/day)	Baseline	4.15 ± 2.55	5.05 ± 5.06	0.031 ^†^ ns ^‡^ ns ^a^
	∆ %	68.32 ± 78.57	44.03 ± 58.17	
Vitamin C (mg/day)	Baseline	129.69 ± 112.03	111.39 ± 77.62	<0.001 ^†^ <0.001 ^‡^ ns ^a^
	∆ %	310.01 ± 159.47	322.48 ± 161.44	

LGI diet—low-glycemic index diet; ST diet—standard diet; ns—not significant (*p* ≥ 0.05); significant differences between baseline and after 8 weeks (Wilcoxon matched-pairs test) at the following groups: ^†^ LGI diet, ^‡^ ST diet; ^a^ significant differences in ∆ % between LGI and ST diet groups (Mann–Whitney U Test; *p* < 0.005).

**Table 7:** Association between the change in DQI scores after 8 weeks of intervention and selected components of nutritional value.

Variable	Spearman’s Correlation with the Change in DQI Score (r) *	
	LGI Diet (*n* = 22)	ST Diet (*n* = 22)
Energy (kcal/day)	−0.51	−0.48
Protein (g/day)	−0.58	ns
Fats (g/day)	−0.64	−0.65
Carbohydrates (g/day)	−0.50	ns
Cholesterol (mg/day)	−0.54	ns
SFA (g/day)	−0.71	−0.62
Fiber (g/day)	ns	ns
Sugars (g/day)	−0.50	ns
Glycemic Load	ns	ns
Sodium (mg/day)	ns	−0.49
Calcium (mg/day)	ns	ns
Vitamin C (mg/day)	0.66	0.62

SFA—saturated fatty acids; LGI diet—low-glycemic index diet; ST diet—standard diet; ns—not significant (*p* ≥ 0.05); * the presented r values for Spearman’s correlation are significant at *p* < 0.05.

## Data Availability

The datasets used and/or analyzed during the current study are available from the corresponding author upon reasonable request. Currently, we do not want to make the data public because it is part of the research for a planned PhD dissertation.

## Abbreviations

The following abbreviations are used in this manuscript:

SFA	Saturated fatty acids
LGI	Low-glycemic index diet
ST	Standard diet
CHILD-2	Cardiovascular Health Integrated Lifestyle Diet-2
DQI	Diet Quality Index
WHO	World Health Organization
BMI	Body mass index
GI	Glycemic index
GL	Glycemic Load
FFQ-6	Food Frequency Questionnaire-6
SD	Standard deviation
RDA	Recommended Dietary Allowance
AI	Adequate Intake
MUFA	Monounsaturated fatty acids
PUFA	Polyunsaturated fatty acids
UPF	Ultra-processed food

## Appendix A

**Table A1:** Assessment of selected components of DQI in the total group (% of participants).

Variable	Intake	Points	Baseline (*n* = 40)	After 8 Weeks (*n* = 40)
Fats (% of energy)	≤30	0	30	60
	30–40	1	65	40
	>40	2	5	0
SFA (% of energy)	≤7	0	10	35
	7–10	1	5	65
	>10	2	85	0
Cholesterol ^1^ (mg/day)	≤200	0	5	15
	200–300	1	35	85
	>300	2	60	0
Protein ^2^ (% of RDA)	≤100	0	0	0
	100–150	1	15	0
	>150%	2	85	100
Sodium ^3^ (% of AI)	≤100	0	15	20
	100–150	1	35	75
	>150%	2	50	5
Calcium ^3^ (% of RDA)	≥100	0	0	5
	67–99	1	15	85
	<67	2	85	10
Whole-grain cereal-based products (portions/day)	≥6	0	5	55
	4–5	1	0	0
	0–3	2	95	45
Vegetables and fruits (portions/day)	≥5	0	85	100
	3–4	1	5	0
	0–2	2	10	0

SFA—saturated fatty acids; RDA—Recommended Dietary Allowance; AI—Adequate Intake; ^1^ adopted recommendations for CHILD-2 [11]; ^2^ intake of total protein in g/kg healthy body weight expressed as % of RDA by gender and age [22]; ^3^ adopted values in accordance with the dietary guidelines for the Polish population by gender and age [22].

**Table A2:** Participant distribution (%) across diet quality categories at baseline and after 8 weeks according to the DQI score in the total group.

	Variable	Baseline (*n* = 40)	After 8 Weeks (*n* = 40)
Diet Quality *			
Unsatisfactory		65	0
Acceptable		30	25
Moderate		5	60
Good		0	15

* The “high” category was not included in the table because no participants met this criterion.

**Table A3:** Mean DQI score before and after nutritional intervention in the total group (mean ± SD).

Variable	Baseline (*n* = 40)	After 8 Weeks (*n* = 40)	*p*-Value * (Wilcoxon Matched-Pairs Test)
DQI	11.25 ± 2.07	6.70 ± 1.20	<0.001

DQI—Diet Quality Index; * significant differences between baseline and after 8 weeks.

**Table A4:** Change (∆ %; mean ± SD) in the nutritional value and glycemic load of the diet after 8 weeks of nutritional intervention in the total group.

Variable		Total (*n* = 40)	*p*-Value * (Wilcoxon Matched-Pairs Test)
Energy (kcal/day)	Baseline	2263.92 ± 562.57	ns
	∆ %	0.84 ± 24.35	
Protein (g/day)	Baseline	94.27 ± 25.81	<0.001
	∆ %	24.70 ± 28.93	
Fats (g/day)	Baseline	81.43 ± 23.21	0.027
	∆ %	−3.87 ± 29.06	
Carbohydrates (g/day)	Baseline	299.81 ± 74.28	ns
	∆ %	0.72 ± 26.79	
Cholesterol (mg/day)	Baseline	372.15 ± 134.44	<0.001
	∆ %	−30.92 ± 26.61	
SFA (g/day)	Baseline	30.54 ± 10.23	<0.001
	∆ %	−31.41 ± 28.86	
MUFA (g/day)	Baseline	30.90 ± 9.12	ns
	∆ %	4.25 ± 32.73	
PUFA (g/day)	Baseline	13.41 ± 6.15	ns
	∆ %	18.29 ±56.46	
*n*-3 (g/day)	Baseline	2.26 ± 1.59	<0.001
	∆ %	89.64 ± 106.49	
*n*-6 (g/day)	Baseline	11.14 ± 5.38	ns
	∆ %	8.26 ± 49.25	
Fiber (g/day)	Baseline	22.57 ± 8.03	<0.001
	∆ %	73.57 ± 53.82	
Sugars (g/day)	Baseline	87.86 ± 35.45	0.007
	∆ %	−7.27 ± 39.20	
Glycemic Load	Baseline	149.39 ± 46.12	<0.001
	∆ %	−23.75 ± 24.20	

* Significant differences between baseline and after 8 weeks (*p* < 0.05); ns—not significant (*p* ≥ 0.05); SFA—saturated fatty acids; MUFA—monounsaturated fatty acids; PUFA—polyunsaturated fatty acids.

**Table A5:** Change (∆ %; mean ± SD) in the mineral levels after 8 weeks of nutritional intervention in the total group.

Variable		Total (*n* = 40)	*p*-Value * (Wilcoxon Matched-Pairs Test)
Sodium (mg/day)	Baseline	2228.10 ± 880.61	<0.001
	∆ %	−14.58 ± 39.14	
Potassium (mg/day)	Baseline	3325.63 ± 790.38	<0.001
	∆ %	49.58 ± 34.37	
Calcium (mg/day)	Baseline	687.36 ± 231.11	<0.001
	∆ %	75.01 ± 87.05	
Phosphorus (mg/day)	Baseline	1350.05 ± 320.51	<0.001
	∆ %	50.66 ± 41.39	
Magnesium (mg/day)	Baseline	293.99 ± 74.70	<0.001
	∆ %	73.88 ± 58.04	
Iron (mg/day)	Baseline	11.75 ± 3.33	<0.001
	∆ %	43.35 ± 39.75	
Zinc (mg/day)	Baseline	11.06 ± 3.15	<0.001
	∆ %	25.90 ± 31.08	

* Significant differences between baseline and after 8 weeks.

**Table A6:** Change (∆ %; mean ± SD) in the vitamin levels after 8 weeks of nutritional intervention in the total group.

Variable		Total (*n* = 40)	*p*-Value * (Wilcoxon Matched-Pairs Test)
Vitamin A (μg/day)	Baseline	1197.83 ± 480.43	<0.001
	∆ %	101.22 ± 77.42	
Vitamin E (mg/day)	Baseline	12.47 ± 5.30	<0.001
	∆ %	83.98 ± 77.31	
Thiamine (mg/day)	Baseline	1.44 ± 0.40	<0.001
	∆ %	29.83 ± 31.03	
Riboflavin (mg/day)	Baseline	1.78 ± 0.55	<0.001
	∆ %	49.47 ± 40.68	
Niacin (mg/day)	Baseline	18.42 ± 6.15	<0.001
	∆ %	55.21 ± 43.90	
Vitamin B_6_ (mg/day)	Baseline	2.13 ± 0.62	<0.001
	∆ %	68.81 ± 46.66	
Folates (μg/day)	Baseline	315.62 ± 121.66	<0.001
	∆ %	103.12 ± 70.51	
Vitamin B_12_ (μg/day)	Baseline	4.55 ± 3.86	0.006
	∆ %	57.39 ± 70.34	
Vitamin C (mg/day)	Baseline	121.45 ± 97.31	<0.001
	∆ %	315.62 ± 158.41	

* Significant differences between baseline and after 8 weeks (*p* < 0.05).

**Table A7:** Association between the change in DQI scores after 8 weeks of intervention and selected components of nutritional value in the total group.

Variable	Spearman’s Correlation with the Change in DQI Score (r) *
Energy (kcal/day)	−0.42
Protein (g/day)	−0.39
Fats (g/day)	−0.53
Carbohydrates (g/day)	−0.38
Cholesterol (mg/day)	−0.35
SFA (g/day)	−0.67
Fiber (g/day)	ns
Sugars (g/day)	ns
Glycemic Load	−0.39
Sodium (mg/day)	−0.40
Calcium (mg/day)	ns
Vitamin C (mg/day)	0.63

SFA—saturated fatty acids; ns—not significant (*p* ≥ 0.05); DQI—Diet Quality Index; * the presented r values for Spearman’s correlation are significant at *p* < 0.05.
